# *Vitreoscilla filiformis* Extract for Topical Skin Care: A Review

**DOI:** 10.3389/fcimb.2021.747663

**Published:** 2021-12-16

**Authors:** Audrey Gueniche, Muriel Liboutet, Stephanie Cheilian, Dominique Fagot, Franck Juchaux, Lionel Breton

**Affiliations:** ^1^ L'Oreal Research and Innovation, Luxury Division Dept, Chevilly-La-Rue, France; ^2^ L'Oreal Research and Innovation, Advanced Research Dept, Aulnay-sous-Bois, France

**Keywords:** *Vitreoscilla*, immunity, skin defences, skin barrier, microbiome

## Abstract

The term probiotic has been defined by experts as live microorganisms, which when administered in adequate amounts, confer a health benefit on the host. Probiotics are, thus, by definition, live microorganisms, and the viability of probiotics is a prerequisite for certain benefits, such as the release of metabolites at the site or adhesion properties, for example. However, some semi-active or non-replicative bacterial preparations may retain a similar activity to the live forms. On cosmetic, lysates or fractions are generally used. Topically applied *Vitreoscilla filiformis* extract has shown to have some similar biological activity of probiotics in the gut, for example, regulating immunity by optimisation of regulatory cell function, protecting against infection, and helping skin barrier function for better recovery and resistance. Due to their mode of action and efficacy, *V. filiformis* extract (lysate including membrane and cytosol) may be considered as non-replicative probiotic fractions, and this review article presents all its properties.

## Introduction

The term probiotic became familiar in 1989 (Fuller, 1989) and was later defined as “live microorganisms, which when administered in adequate amounts, confer a health benefit on the host” by the Food and Agriculture Organization of the United Nations and the WHO (FAO/WHO, 2001) and adopted by the International Scientific Association for Probiotics and Prebiotics (Hill et al., 2014). Indeed, the benefits for human health of consuming fermented foods have been known for centuries, long before the discovery of microorganisms. Over a century ago, Metchnikoff introduced the idea that modifying the intestinal microbiome by the administration of host-friendly bacteria found in yoghurt might confer a positive health benefit and delay senility (Gordon, 2008).

Probiotics contribute to maintaining homeostasis and prevention and/or treatment of host, including (Khare et al., 2018; Sanders et al., 2018; Fang and Brent Polk, 2020; Daliri et al., 2021).

1. Blocking pathogenic bacterial effects by producing antibacterial substances and competing with pathogens for binding to epithelial cells
2. Promoting intestinal epithelial cell homeostasis by increasing barrier function, mucus production, survival, and cytoprotective responses
3. Defining the balance between necessary and excessive defence immunity by increasing innate immunity, such as production of IgA and defensin, upregulating anti-inflammatory cytokine production, and inhibiting proinflammatory cytokine production
4. Regulating the gut–brain axis through the production of neurotransmitters and through the vagus nerve.

Specific strains of probiotic lactic acid bacteria can beneficially influence the composition and/or metabolic activity of endogenous microbiota (Langhendries et al., 1995; MacFarland, 1999; Isolauri et al., 2001; Mohan et al., 2006), and some have been shown to inhibit the growth of a wide range of entero-pathogens (Bernet-Camard et al., 1997; Coconnier et al., 1998). Competition for essential nutrients or for receptor sites (Coconnier et al., 1993), aggregation with pathogenic microorganisms (Rolfe, 2000), and the production of anti-microbial metabolites (Bernet-Camard et al., 1997; Coconnier et al., 1998) have all been reported to play a role.

Beyond their capacity to positively influence the composition of intestinal microbiota (Midolo et al., 1995; Roberfroid et al., 1995; Benno et al., 1996; Cebra, 1999; Pochapin, 2000; Ouwehand et al., 2003; Bergonzelli et al., 2005; Sarker et al., 2005), evidence suggests that some probiotic bacteria can modulate the immune system at both the local and systemic levels (Cebra, 1999; Isolauri et al., 2001; Magerl et al., 2008; Benyacoub et al., 2014; McFarland et al., 2018), thereby improving immune defence mechanisms and/or downregulating immune disorders (Isolauri, 2001; Kalliomäki et al., 2001; Rautava and Isolauri, 2002). Several strains of lactic acid bacteria were shown to modulate cytokines and growth factor production *in vitro* and *in vivo* (von der Weid et al., 2001; Christensen et al., 2002; Borruel et al., 2003). Moreover, oral probiotics have been shown to play a protective role in epithelial barrier function and wound healing, and this can occur through direct action at the level of the gut (Rao and Samak, 2013) but also indirectly at a distance beyond the gut, for example, in the skin (Benyacoub et al., 2014).

The ability of probiotics to confer epithelial protection, immunoregulation, and barrier function recovery have been used for:

- Treating intestinal diseases such as gastroenteritis (Szajewska et al., 2014), intestinal hyper-permeability (White et al., 2006), intestinal dysbiosis (Schepper et al., 2019), irritable bowel syndrome (Moayyedi et al., 2010), Crohn’s disease (Guslandi et al., 2000), colon cancer (Drago, 2019), ulcerative colitis (Dhillon and Singh, 2020), and peptic ulcer (Ameen et al., 2019)
- Decreasing hypertension and serum cholesterol levels in cardiovascular disease (Liu et al., 2017)
- Modulating pain sensations by inducing opioid and cannabinoid receptor expression and serotonin precursors (Ringel-Kulka et al., 2014)
- Preventing and/or treating allergic rhinitis (Du et al., 2019)
- Treating several skin conditions to prevent and reduce the severity of, for example, atopic dermatitis (AD) (Li et al., 2019), dandruff (Reygagne et al., 2017), sensitive skin (Gueniche et al., 2014), and dry skin; protecting against photo immunosuppression (Peguet-Navarro et al., 2008); and facilitating wound healing (Han et al., 2020)

Uses based on the metabolic properties of probiotics require live probiotic forms; however, some semi-active or non-replicating bacteria preparations may also retain activity comparable with that of the live forms (Mathur et al., 2020). Such semi-active and non-replicative bacterial forms are of interest for topical preparations designed for skin, beauty, and health purposes (Rodrigues et al., 2005; Sawada et al., 2007; Gueniche et al., 2010; Lopes et al., 2017; Jung et al., 2019; Lim et al., 2020; Brandi et al., 2020; Franca, 2021).

In cosmetic non-replicative probiotic cells, their lysates or fractions are generally used.

Here, we review the mode of action and efficacy of *Vitreoscilla filiformis* extract (Vfe; lysate including membrane and cytosol) that demonstrate that this ingredient may be considered as non-replicative probiotic fractions.

## *Vitreoscilla filiformis* Extract

*V. filiformis* (ATCC 15551) is a non-photosynthetic, non-fruiting, filamentous, bacterium belonging to the Beggiatoales order, as classified according to Bergey’s manual (Bergey and Holt, 1994); it was named on the basis of its colourless (“vitreo” glass-like and transparent), gliding filamentous morphology (“filiformis”). This gram-negative non-pathogenic bacterium was isolated by Dr Joseph Victor Jullien from sodium sulfurised thermal waters at a spa resort in the Pyrénées-Orientales, France. The anti-pruritic and anti-inflammatory properties of the thermal water are well known, and the dermatological spa therapy is prescribed for patients with AD and psoriasis.

The complete genome sequence of *V. filiformis* is an assembled genome of one chromosome and two plasmids (Strohl et al., 1986; Schmidt et al., 1987; Stahl et al., 1987; Contreras et al., 2017). The extract is very rich in amino acids, including all 13 non-essential amino acids currently known and all the nine essential amino acids that cannot be synthesised *de novo* by human metabolism.

Vfe (also called pure extract) is a natural, 100% biodegradable ingredient that has been produced in a unique sustainable process. Vfe has been produced on a large scale for over 20 years by an exclusive, green, bio-fermentation process to generate a bacterial lysate. The well-defined, sterile culture medium contains autolytic yeast extract (2 g/L), soya papain peptone (2 g/L), glucose (3 g/L), Heller microelements (1 ml/L), and CaCl_2_*2 H_2_O (60 mg/L), at pH 7.2. The bioreactor medium is inoculated with *V. filiformis* (20 ml/L). After being harvested, Vfe is separated from the culture medium by centrifugation at 10,000 *g* for 10 min, and the biomass is stabilised by heat treatment at 121°C for 30 min.

This biomass, marketed as Vfe, is used as an industrial raw material in many cosmetic preparations. Vfe has a similar mode of action when applied topically on the skin as that of probiotics in the intestine (regulation, protection, and repair), including the ability to modulate immunity, decrease inflammation, stimulate skin defences, and improve the skin barrier, as described below.

## Skin Benefits of *Vitreoscilla filiformis* Extract

### Immunoregulation/Soothing Effect

The innate immune system activates the adaptive response (cytokine/chemokine release or co-stimulatory signals expressed by antigen-presenting cells [APCs]), by recognition of molecules on or produced by microorganisms. These pathogen-associated molecular patterns (PAMPs) are recognised by specific host receptors, especially toll-like receptors (TLRs) and nucleotide-binding oligomerisation domain (NOD) receptors (Medzhitov and Janeway, 2000). TLRs are expressed at the surface of monocytes, macrophages, and dendritic cells (DCs). DC maturation represents a bridge between the innate and adaptive immunity systems and depends on the binding of PAMPs to TLRs expressed at their surface.

TLRs play an essential role in immune development into conventional T helper (Th) for humoral immune response (Th2), cellular immune response (Th1), or regulatory T cells (Tr1, Th3, and CD4+CD25+) responses according to the type of stimulation (Amati et al., 2006).

TLR2 predominantly recognises Gram-positive bacterial components, especially from probiotic lactobacilli, such as peptidoglycans. TLR4 recognises bacterial endotoxin lipopolysaccharides (LPSs), which constitute the major component of the outer membrane of Gram-negative bacteria (Mukhopadhyay et al., 2004).

The epidermis also plays an important role in innate immune responses through epidermal keratinocyte TLR activation. It is now well established that the TLR2 subtype present in keratinocytes responds locally to initiate the immune pathway and thus may recognise commensal microorganisms and probiotics (Medzhitov and Janeway, 2000; Kawai et al., 2002; Pivarcsi et al., 2003). However, no chemical compounds or biotechnological biomass (or constituents) have yet been identified that induce the TLR2 pathway locally in the skin.

TLR2 activation by bacteria can result in either inflammatory or tolerogenic immune responses (Oliveira-Nascimento et al., 2012). It has been demonstrated for *Neisseria meningitidis*, which is a closely related Gram-negative bacteria within the Neisseriaceae family as *V. filiformis*, that the TLR2 activating ligand is a bacterial porin with anti-inflammatory properties (Strohl, 2005).

**Figure 1:**
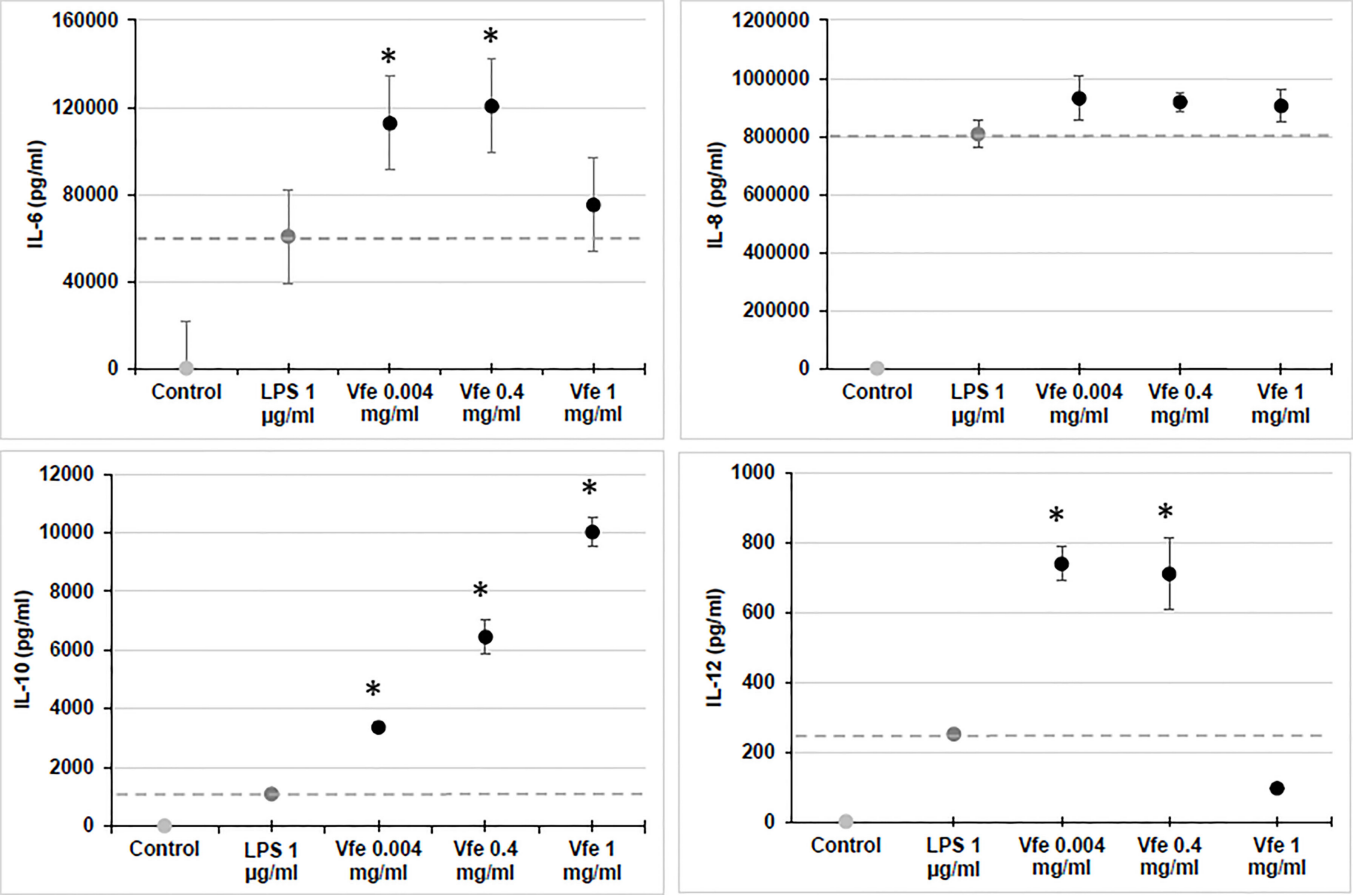
Stimulatory effect of *Vitreoscilla filiformis* extract (Vfe) on cytokines in monocytes (mean ± SEM). The effect of Vfe 0.2% (black circles) on cytokine stimulation *in vitro* was evaluated in monocytes isolated from peripheral blood mononuclear cells and compared with lipopolysaccharide (dark grey circle). Monocytes were incubated with the test compounds for 36 h, and the supernatant was collected and assayed for cytokines (IL-6, IL-8, and IL-10 and IL-12p40) using the Milliplex assay kit and Luminex LX100 apparatus. *p < 0.05 compared with lipopolysaccharide (LPS).

In contrast to Gram-negative pathogenic bacteria eliciting proinflammatory immune responses, innate immune sensing of non-pathogenic Gram-negative bacteria like *V. filiformis* was characterised by TLR2 signalling over TLR4 signalling (Volz et al., 2014). Similar to the way in which oral probiotics act on DC in the gut, Vfe regulates skin homeostasis by predominantly inducing the release of interleukin-10 (IL-10) through the TLR2 pathway in DC. The release of IL-10 leads to a very high IL-10/IL-12 ratio for an optimal Th1/Th2 balance and induction of T-regulatory cell function (**Figure 1**). Most of the following properties are maintained in TLR4 knock-out cells, demonstrating that the observed efficacy is independent of the LPS of Vfe (Volz et al., 2014).

These pathways to induce tolerance may help explain the efficacy of Vfe on AD prone skin that was demonstrated in three proof-of-concept studies (Gueniche et al., 2006; Gueniche et al., 2008b; Gueniche et al., 2008c).

Interestingly, *in vitro* experiments also demonstrated that Vfe (0.033%–0.3%) significantly stimulated the chemotactic migration of polymorphonuclear neutrophils using a Boyden chamber assay in a dose-dependent manner and significantly increased the phagocytosis of monocytes and Langerhans cells using fluorescent beads. Monocytes freshly isolated from whole blood and Langerhans cells were seeded in a 96-well plate in culture medium with or without (control) Vfe. The phagocytosis of fluorescent beads was calculated by gating and analysing fluorescein isothiocyanate (FITC)-positive (fluorescent) cells. Vfe significantly increased the phagocytosis of Langerhans cells 1% (48.2% ± 8.0% compared with 14.2% ± 2.3% of FITC-positive cells; p < 0.01) and monocytes 0.3% (74.1% ± 1.3% compared with 45.4% ± 2.1% of FITC-positive cells; p < 0.01).

Vfe was also shown to stimulate the expression of TNFAIP3 (A20) in human epidermal keratinocytes. Since TNFAIP3 (A20) regulates proinflammatory genes link to NFkb, it indicates an anti-inflammatory activity (Urbano et al., 2018).

Furthermore, inflammation induced by *Cutibacterium acnes* ATCC^®^6919, as measured by stimulation of IL-8 secretion on human sebocytes (2,206 vs. 45 pg/ml), was significantly inhibited in the presence of 2 μg/ml of Vfe (1,195 vs. 2,206 pg/ml).

In recent *in vitro* studies to evaluate the resolutive pathways, Vfe significantly stimulated lipoxins A4 and B4, protectins D1 and DX, resolvins D4 and D5, and resolvins E1 and E2 and decreased thromboxane B2, prostaglandin E2, and leukotriene B4 (**Table 1**). This efficiency in stimulating pro-resolution is a fundamental property especially during chronic inflammatory processes (Serhan and Levy, 2018; Sugimoto et al., 2019; Fu et al., 2020).

**Table 1:** Pro-resolving effect of Vfe.

% activation stimulated with PMA + A23187	Control DMSO 0.1%	Vfe 0.4 mg/ml
Lipoxin A4	0	43
Protectin D1	0	79*
Protectin DX	0	96*
Resolvin D5	0	188*
Resolvin E1	0	45
Resolvin E2	0	22
Thromboxane B2	0	142*
5-Hydroxyeicosatetraenoic acid	0	−24
Prostaglandin E2	0	913*
Leukotriene B4	0	68

In vitro model including co-cultured primary human keratinocytes and dendritic cells. Vfe was pre-incubated with the cells before induction of inflammation using phorbol myristate acetate (PMA) + calcium ionophore A23187, and lipid mediators were quantified using liquid chromatography coupled to tandem mass spectrometry.

Vfe, Vitreoscilla filiformis extract; DMSO, dimethyl sulfoxide.

*p < 0.05.

In order to demonstrate the protective activity of the Vfe against irritation induced by methyl nicotinate, blood microcirculation was analysed using Doppler laser on ten subjects of mean age of 28 years. In the area treated with Vfe, there was a significantly smaller increase in blood flow following the application of methyl nicotinate compared with placebo.

Vfe has also been shown to reduce gene expression of thymic stromal lymphopoietin receptor (TSLPR), IL31R and cytokines TSLP, IL-31, which are stimulated by LPS, lipoteichoic acid, and zymosan, which are used to mimic pathogenic colonisation in keratinocytes, endothelial cells, or T cells (Kempkes et al., 2012). IL-31 is a T cell-derived cytokine linked to pruritus in skin inflammation, while TSLP is an excellent candidate for mediating the innate immune response triggered by viruses or bacteria. All these results may explain the efficacy of Vfe in reducing pruritus of seborrheic dermatitis or AD (Gueniche et al., 2008a).

Vfe stimulated *in vitro* sensory neurons cultured by a modified reported method with and without capsaicin decreased calcitonin gene-related peptide (CGRP) (**Table 2A**). In addition, the effect of Vfe on substance P-induced neurogenic skin inflammation was tested *ex vivo* in human abdominal plastic skin explant models. In the positive control, substance P stimulated a statistically significant increase in vasodilation, oedema, and mast cell degranulation. These substance P-induced vasodilation and oedema were abrogated by the presence of Vfe (**Table 2B**). Also, mast cell degranulation induced by substance P, as measured by CGRP liberation after capsaicin stimulation of sensitive neurons, was significantly lower in the presence of Vfe compared with the control (**Table 2C**).

**Table 2:** Effect of Vfe on *in vitro* models of sensitive skin.

(A)		
Treatment	%	
	15-min incubation	6-h incubation
Control medium	100	100
Vfe 0.1%	90	70 (p < 0.01)
Control medium + capsaicin	100	100
Vfe 0.1% + capsaicin	74 (p < 0.01)	69 (p < 0.01)
**(B)**		
**Treatment**	**% Dermal capillaries dilated**	
Control skin	64.45 ± 12	–
Skin + substance P	89.2 ± 6.3	p < 0.01 vs. control skin
Skin + substance P + Vfe 0.1%	69.6 ± 14.9	p < 0.01 vs. skin stimulated by substance P
**(C)**		
**Treatment**	**Dermal oedema score**	
Control skin	0.86 ± 0.7	–
Skin + substance P	1.9 ± 0.7	p < 0.05 vs. control skin
Skin + substance P + Vfe 0.1%	1.05 ± 0.7	p < 0.05 vs. skin stimulated by substance P

Spontaneous and capsaicin-induced release of calcitonin gene-related peptide by sensory neurons stimulated by Vfe (A), and using an alternative method of substance P-induced neurogenic inflammation of the skin (Boisnic et al., 2003) to evaluate of the effect of Vfe on the dilation of dermal capillaries (B) and on dermal oedema (C).

Vfe, Vitreoscilla filiformis extract.

The efficacy against neurogenic inflammation has been validated by results from stinging tests in a clinical trial in volunteers with sensitive skin. After 4 weeks of daily applications, the sensitive skin was significantly less reactive to lactic acid, showing a significant soothing effect over time for the Vfe formula (Li et al., 2006).

### Natural Defences/Anti-Oxidant Effects

The human skin forms the interface between the body and outside environment and is endowed with innate immune defence mechanisms against infection. The epidermis (epithelial skin compartment), which covers the surface of the whole body, acts as a mechanical barrier and frontline of defence against pathogenic microbial invasion. It is an active immune organ playing a crucial role in innate immune responses that protects the body against microbial invasion by killing pathogenic microorganisms through the production of cationic antimicrobial peptides (AMP), such as human β-defensins and cathelicidin (Harder et al., 2004; Braff et al., 2005; Sumikawa et al., 2006). It also provides a home to the commensal microbiota and regulates the microbial balance of these complex microbial communities (Grice et al., 2009; Grice and Segre, 2011). In addition, the skin microbiome plays an integral role in the maturation, protection of the skin barrier function, and homeostatic regulation of keratinocytes and host immune networks; thus, it is fundamental to keep it well balanced (Suter et al., 2009; Chen et al., 2018).

β-Defensin 2 and S100A7 are AMPs that are able to decrease the growth of *Malassezia restricta* and *Malassezia globosa* fungi, as well as Gram-negative and Gram-positive bacteria. In keratinocytes (Vfe 0.3%) and reconstructed epidermis (topically applied Vfe 10%), Vfe significantly stimulated β-defensin 2 and S100A7 at the gene expression and protein levels (specific PCR, immunolabelling, and ELISA dosage) by 7,500 vs. 0 pg/ml and 50 vs. 25 ng/ml, respectively.

**Figure 2:**
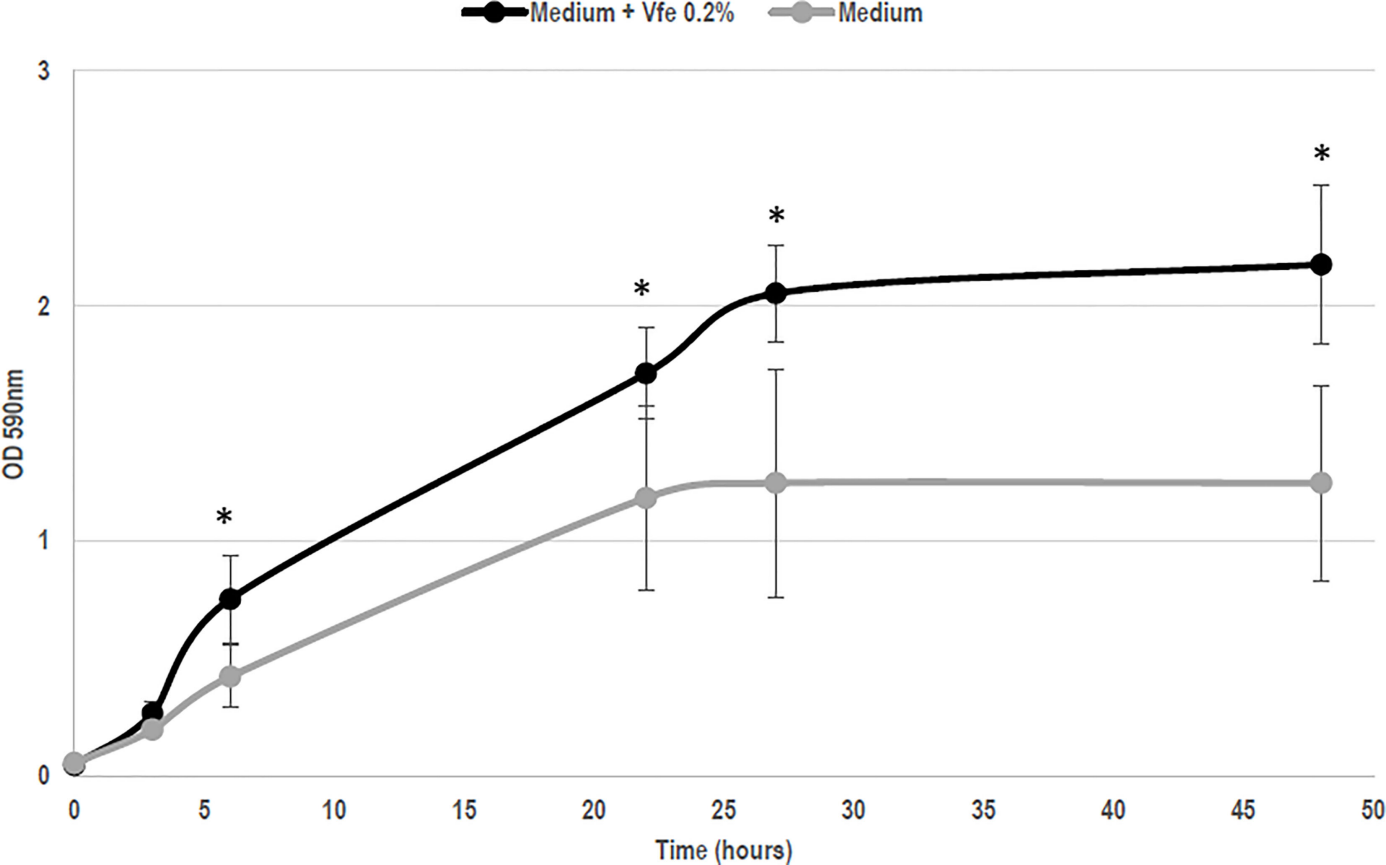
*Vitreoscilla filiformis* extract (Vfe) induces growth enhancement of *Staphylococcus epidermidis* (mean ± SD). In an *in vitro* method based on growth kinetics, bacteria were incubated with Vfe in a liquid culture of a minimal medium composed of glucose, potassium phosphate, ammonium sulfate, heptahydrate magnesium sulfate, and NaCl, at pH 6. The presence of Vfe at 0.2% significantly enhanced the growth of *S. epidermidis* compared with the conditions without Vfe. *p < 0.05 compared with medium alone.

Although Vfe does not kill bacteria or fungi, Vfe was recently shown to specifically stimulate the growth of *Staphylococcus epidermidis*, one of the main beneficial bacteria (**Figure 2**). This finding demonstrates that it might be of interest on skin health.

In addition to the direct immunomodulatory effect of Vfe on skin-associated immune responses, the stimulation of epidermal AMP and growth of *S. epidermidis* may help to rebalance the skin microbiota due to a reduction in *S. aureus*, which may partly explain the beneficial effects of Vfe on AD (Gueniche et al., 2008c).

*In vitro* studies using keratinocytes and fibroblasts have shown that Vfe stimulates endogenous anti-oxidant defences by stimulating mitochondrial manganese superoxide dismutase 2 (MnSOD2) activity at both the mRNA and protein levels (Mahé et al., 2006; Mahe et al., 2013). These results suggest that Vfe could induce skin cells to produce their own endogenous protective defences against both exogenous environmental stressors, such as UV radiation, as well as to combat endogenous sources of deleterious free radicals involved in skin ageing. Furthermore, Vfe was found to significantly inhibit the appearance of sunburn cells in UVB‐exposed areas, a signature of skin alteration that may be linked to a defect in MnSOD protective activity (Mahé et al., 2006; Mahe et al., 2013).

**Figure 3:**
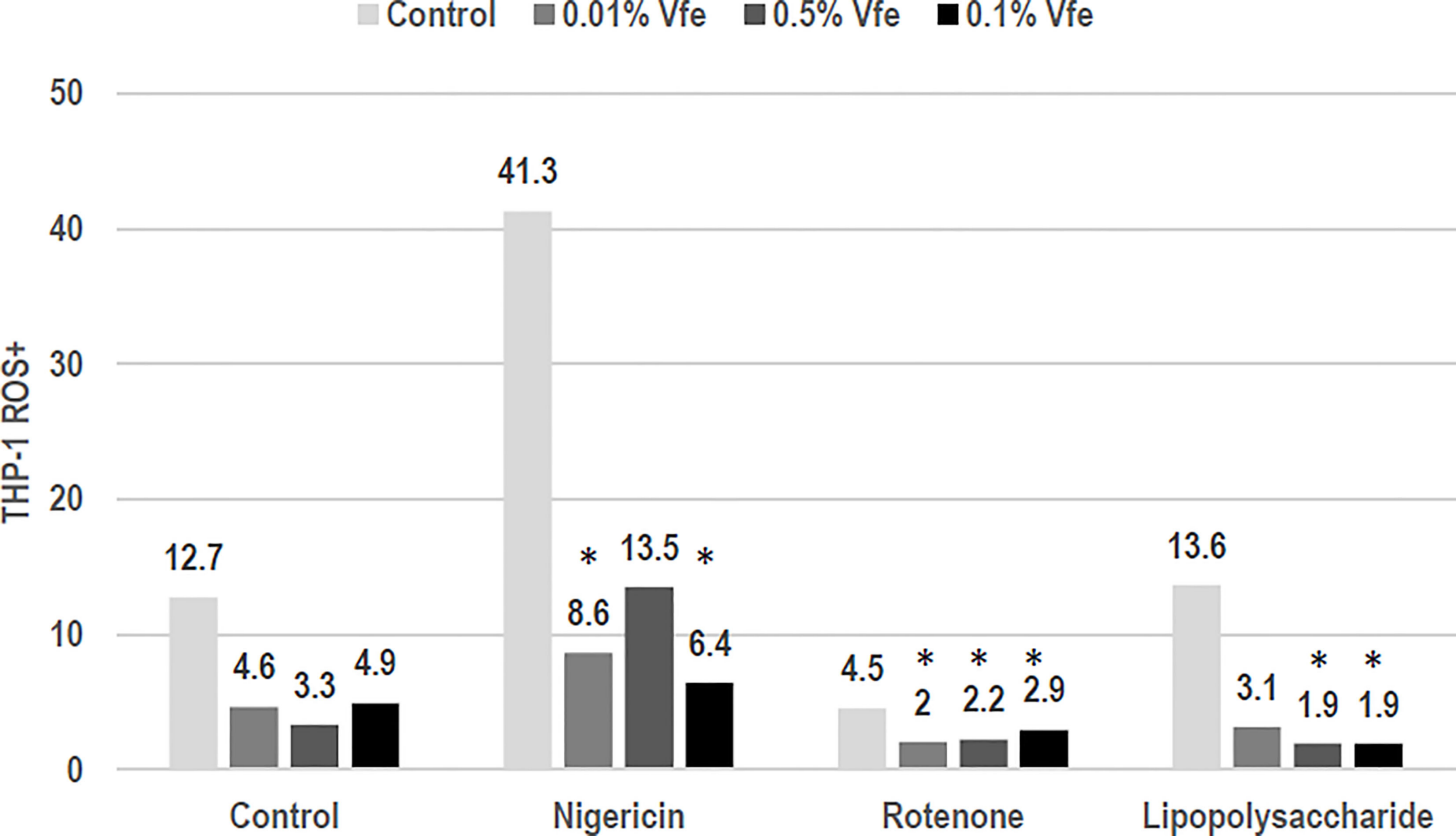
Inhibitory effect of *Vitreoscilla filiformis* extract (Vfe) on the inflammasome. In an *in vitro* model of the NLRP3 inflammasome on monocytes (THP-1 cell line), the inflammasome was activated by uncoupling the mitochondrial respiratory chain, causing the production of reactive oxygen species (ROS). ROS were induced by nigericin, rotenone, or the control lipopolysaccharide. The percentage of ROS+ cells was defined according to flow cytometry mitotracker markers. The addition of Vfe decreased the percentage of ROS+ cells. *p < 0.05 compared with control.

Microbial stress from PAMP or other damage-associated molecular patterns induces a decoupling of the mitochondria that results in the release of reactive oxygen species (ROS). These ROS are then capable of activating the inflammasome, causing the pro-cytokine precursors to mature into effective and inflammatory cytokines. Vfe showed a dose–response decrease in mitochondria uncoupling following microbial stress and thus protected the respiratory chain, significantly limiting the release of ROS. Moreover, this dose-dependent activity was maintained when the number of altered mitochondria was increased by a respiratory chain uncoupling agent, particularly on complex III (CoQ target) such as rotenone (**Figure 3**). This unique activity, to reduce the number of altered mitochondria and thus the quantity of intracellular ROS, is important as the activation of the inflammasome NLRP3 can be induced by skin microbiota dysbiosis, for example, an imbalance of *Malassezia* spp. during seborrheic dermatitis leading to inflammation (Gueniche et al., 2008a; Kistowska et al., 2014).

CD59 protectin is a protective shield that protects the membranes of cutaneous cells against non-specific cell lysis. In keratinocytes incubated with Vfe 0.1%, CD59 protectin mRNA expression was stimulated 4.9 times (quantitative RT/PCR) and CD89 protectin levels increased by 1.8 times (p < 0.01) (fluorescence cell sorter analysis). When keratinocytes were incubated at 37°C for 24 h without thermal stress, Vfe did not induce the expression of HSP70. However, 30 min after being subjected to thermal stress, Vfe 0.2% stimulated the production of HSP70 by 500% to 700% compared with control keratinocytes, indicating that it stimulates cellular defences.

Several epidemiological studies have demonstrated that exposure to UV induces dramatic changes in immune functions. Among these changes, a decrease in the number and morphological modifications of Langerhans cells, as well as an alteration in their capacity to present antigens, has been demonstrated. Vfe was found to protect Langerhans cells in the reconstructed epidermis or skin explants exposed to UV, indicating preservation of the skin immune homeostasis even when exposed to solar radiation (**Table 3**).

**Table 3:** Effect of *Vitreoscilla filiformis* extract 0.1% on Langerhans cells in skin explants exposed to UVA/UVB irradiation.

Exposure	Treatment	% Positive cells	p-Value
No UV	Control	100	
UV exposure	Control	0	p < 0.01 vs. control without UV
UV exposure	SPF20 sun cream	62	p < 0.01 vs. control UV
UV exposure	Vfe 100%	70	p < 0.01 vs. control UV
UV exposure	Vfe 50%	61	p < 0.01 vs. control UV

Skin explants were topically pre‐treated with Vfe (5 mg/cm^2^) or the reference (solar cream, 5 mg/cm^2^) or left untreated (control). After 24 h pre‐incubation, the treatment was renewed, and the skin explants were incubated for 45 min before being exposed to UVB 1.25 J/cm² (and UVA 3.8 J/cm²) BS‐02 (Opsytec Dr. Gröbel) or kept in the dark (unexposed control). Immediately after UV exposure, the treatment was renewed, and skin explants were incubated for 24 h. After incubation, the skin explants were rinsed, and 8-mm punches were taken for immunolabelling. The frozen sections were incubated for 1 h with the appropriate primary antibody (anti‐CD1a, BD Biosciences, ref. 555805), the binding sites were revealed using the appropriate secondary antibody, and the cell nuclei were stained with propidium iodide (PI; Sigma, ref. P4170) solution.

### Strengthening the Skin Barrier Function and Skin Integrity

Skin barrier structure and function are essential to human health. The most important function of the skin is to act as a barrier between the “inside” and the “outside” of the organism to prevent invasion of pathogens and fend off chemical assaults, as well as the unregulated loss of water and solutes. The *stratum corneum*, which consists of protein-enriched cells and lipid-enriched intercellular domains, is the main physical barrier. Any modifications in epidermal differentiation and lipid composition result in altered barrier function, a central event in various skin alterations and diseases.

**Figure 4:**
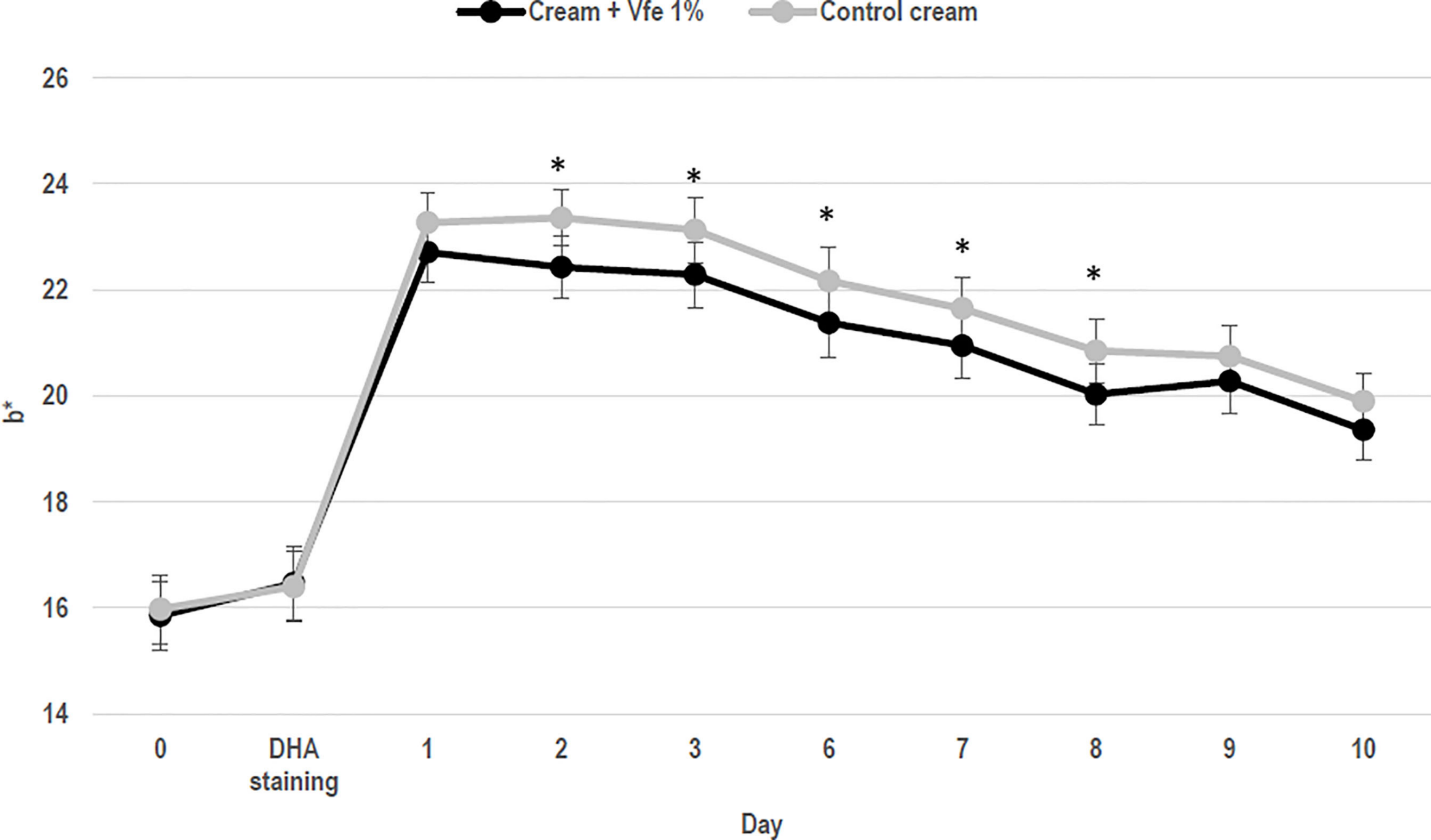
Stimulatory effect of *Vitreoscilla filiformis* extract (Vfe) on skin cell renewal rate *in vivo* (mean ± SD). In 22 women, volunteers aged from 24 to 55 years, the effect of Vfe 1% versus a control formula on the cell renewal rate after 2 weeks of pre-treatment and 10 days of treatment was evaluated by the decrease in skin colour after staining using 10% dihydroxyacetone (DHA) cream, as measured by Chroma meter CR400. Parameter b* values before and after treatment showed that the DHA-induced colour decreased significantly more rapidly on the arm treated with the formula containing Vfe 1% than on the arm treated with the vehicle cream. *p < 0.05 compared with vehicle cream.

Keratinocyte growth was evaluated by measuring 3H-thymidine incorporation in DNA. When cells were treated with Vfe, an increase in 3H-thymidine incorporation was observed demonstrating that Vfe increases keratinocyte proliferation. The turnover time of the stratum corneum is an important parameter since it is linked to epidermal cell turnover, which decreases with age. The *in vitro* efficacy was corroborated by clinical studies evaluating the epidermal turnover. *In vivo*, the application of Vfe significantly increased the *stratum corneum* renewal rate, as measured by a faster reduction in dansyl chloride fluorescence, compared with the control skin. The mean cell turnover was faster for Vfe treated skin (17.3 ± 3.4 days) compared with the untreated skin (20.7 ± 2.2 days; p < 0.0001), and the mean area under the curve was lower at 57.2 vs. 66.8, respectively (p < 0.0001). The cell renewal rate was also significantly faster in the presence of Vfe when measured by the decrease in colour from staining with 10% dihydroxyacetone (DHA) (**Figure 4**).

Vfe 1% induced faster *in vitro* keratinocyte (monolayers seeded on collagen 1) migration than the control with better efficacy in reepithelisation (297 ± 84 migrative cells compared with 201 ± 28 for the control). Additionally, using a new *in vitro* epidermal regeneration 3D model for keratinocyte migration, mimicking phase II of wound healing (Deshayes et al., 2018), Vfe significantly increased wound healing at day 16 (Vfe 0.3%) and day 17 (Vfe 0.1% and 0.3%).

The adherens and tight junctions in the epidermis, in addition to the abundance of corneodesmosome, play a fundamental role in skin physiology by mediating firm mechanical stability between the cells. Indeed, these structures are crucial components of the skin barrier function and link structural integrity to proliferation and inflammatory responses in the skin. Tight junctions in the stratum granulosum regulate “apical” protein and lipid vesicle targeting toward the stratum corneum. Vfe was shown to increase the quantity of zonula occludens-1 in normal keratinocytes, which is one of the main tight junction proteins (198% increase with Vfe 0.05% and 354% increase with Vfe 2% compared with 340% for the positive control CaCl_2_, 1.5 mM).

Each maturation step contributing to the formation of an effective moisture barrier, especially corneocyte strengthening, lipid processing, and natural moisturising factor generation (a complex mixture of low-molecular-weight, water-soluble compounds formed within the corneocytes by degradation of filaggrin), is influenced by the level of stratum corneum hydration. Inefficient degradation of corneodesmosomes and accumulation of corneocytes on the skin’s surface cause dry, flaky, and dull-looking skin. In normal human keratinocytes, Vfe was shown to increase the expression of loricrin (a protein precursor of the stratum corneum) and three proteins fundamental for differentiation, including transglutaminase (epidermal TGK are involved in cornified envelope formation by cross-linking structural proteins), LEP16 (late envelope proteins are linked to cornified envelope proteins), and corneodesmosin (a protein found in corneodesmosomes required for desquamation) (**Table 4**).

**Table 4:** Effect of Vfe on normal human keratinocytes and fibroblasts.

		Expression % of control	p-Value
**Transglutaminase**	Control	100	
	Vfe 1%	119	*
	Vfe 0.1%	120	*
**Filaggrin**	Control	100	
	Vfe 0.5%	147	*
**LEP16**	Control	100	
	Vfe 1%	8,573	***
	Vfe 0.2%	898	**
**Corneodesmosin**	Control	100	
	Vfe 1%	445	**
	Vfe 0.2%	317	**
**Loricrin**	Control	100	
	Vfe 0.002%	259	***
	Vfe 0.001%	189	**
	Vfe 0.0002%	151	
**Collagen I**	Control	100	
	Vitamin C + Transforming growth factor-β	372	***
	Vfe 0.05%	146	**
	Vfe 0.017%	127	***
**Collagen IV**	Control	100	
	Vitamin C + Transforming growth factor-β	2,937	**
	Vfe 0.05%	288	**
	Vfe 0.017%	136	

Gene expression for transglutaminase and differentiation markers in keratinocyte monolayers and on collagens I and IV in fibroblast monolayers using immunolabelling.

Vfe, Vitreoscilla filiformis extract.

*p < 0.05; **p < 0.01; ***p < 0.001.

**Figure 5:**
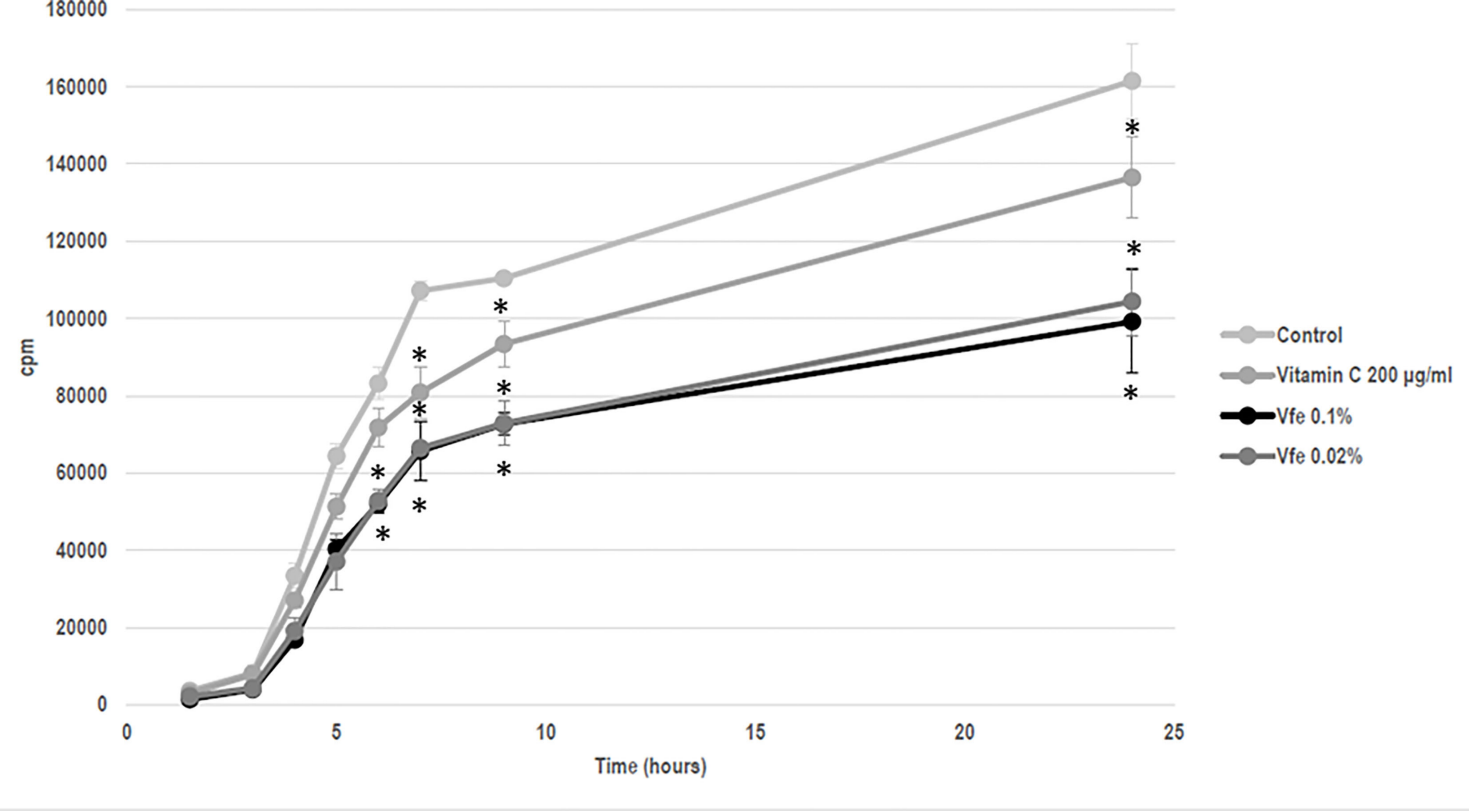
*Vitreoscilla filiformis* extract (Vfe) decreased the diffusion rate of caffeine through reconstructed skin (mean ± SD). A 13-day culture of EPISKIN™ was incubated for a total of 5 days with Vfe, vitamin C reference, or the control. The epidermis was then washed and 100 μl of 2 μCi/ml (0.04 mM) radioactive caffeine (14C-caffeine, Perkin Elmer Ref. NEC41205UC) and 0.35 mM of cold caffeine (Sigma C8960), corresponding to around 500,000 cpm in total, was applied to the surface of each epidermis before media sampling kinetic measurements. The treatment by Vfe 0.2% and 0.04% decreased the rate of caffeine diffusion by 40% to 50% compared with the control. *p < 0.05 compared with control.

**Figure 6:**
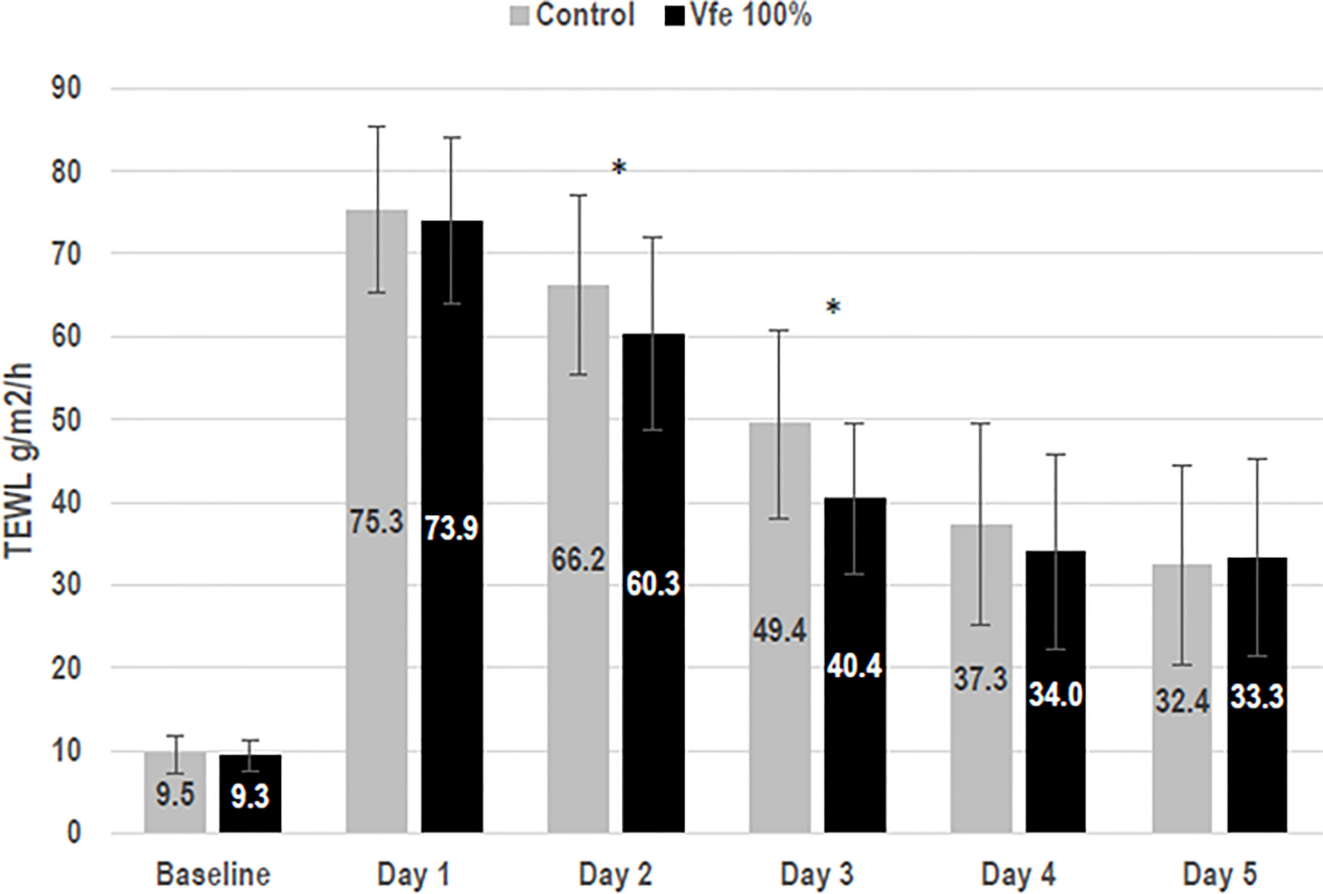
*Vitreoscilla filiformis* extract (Vfe) increased skin barrier recovery rates after tape stripping (mean ± SD). To evaluate skin barrier recovery rates in female volunteers aged 25 to 55 years, test areas on the inner forearm were subject to barrier damage by tape stripping using D-Squames^®^ on day 1. Vfe was then applied twice daily to the test area for 4 days or left untreated (control). Skin barrier recovery was assessed by measuring on days 1 to 5. As expected, tape stripping impaired the skin barrier, as shown by increased TEWL. Natural barrier recovery was observed for the untreated control area between days 2 and 5 compared with day 1. TEWL, transepidermal water loss. *p < 0.05 compared with control zone.

The efficacy of Vfe on reinforcing the skin barrier function was shown *in vitro* using reconstructed skin. The rate of diffusion of caffeine through reconstructed skin was slowed after the application of Vfe, indicating an improvement in skin barrier function (**Figure 5**). Furthermore, Vfe improved barrier function recovery *in vivo* after tape stripping compared with the control area, as evaluated by Tewameter^®^ on healthy volunteers (**Figure 6**). A significantly better improvement of skin barrier was found after 1 (p = 0.017) and 2 days (p < 0.001) of treatment with Vfe compared with the control skin that was left untreated after tape stripping.

Laminin 5 is found in the hemidesmosome adhesive junction and contributes to the cohesion of epithelial cells for a strong dermal–epidermal junction (DEJ), while perlecan is a proteoglycan found in the DEJ and is able to store and protect cellular growth factors. The application of Vfe 0.05% stimulated perlecan 180% with taking the control as 100%. Also, Vfe had a protective effect on laminin 5 and perlecan altered by corticoids in *ex vivo* skin.

**Figure 7:**
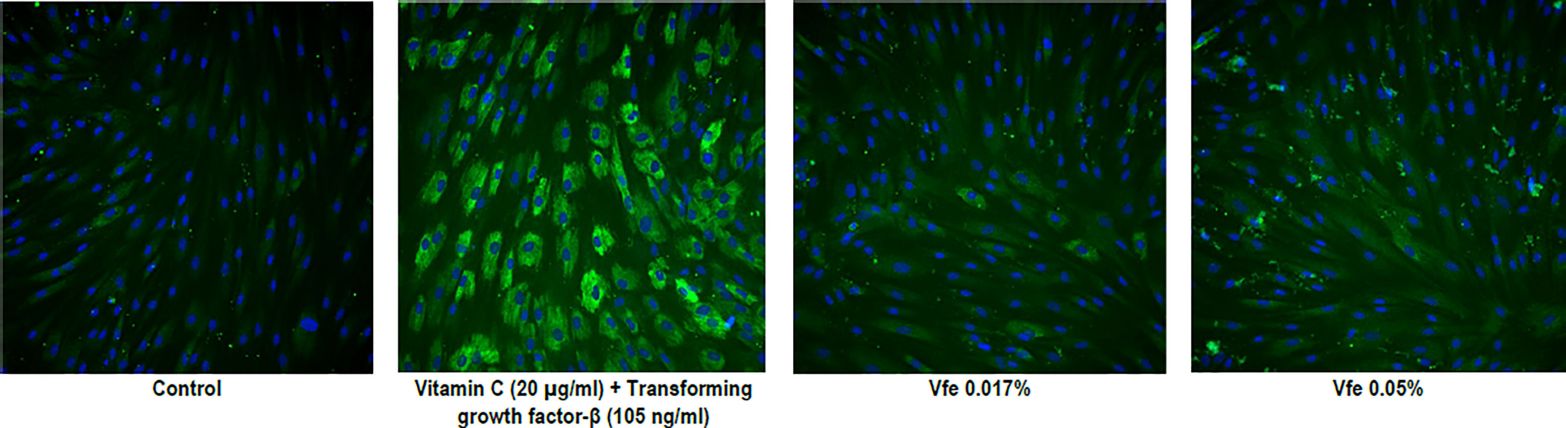
Stimulatory effect of *Vitreoscilla filiformis* extract (Vfe) on collagen IV expression in human fibroblasts. Representative high-resolution images of *in situ* immunolabelling demonstrating the significant effect of Vfe (0.017% and 0.05%) on collagen IV expression in human fibroblasts.

Finally, type I and IV collagens are important constituents of the skin. High proportions of type I collagen are found in all dermal layers, while type IV collagen is localised in the basement membrane of the DEJ. Although they possess closely related amino acid compositions, they present important structural differences that confer specific biophysical properties. When the expression of proteins was measured in normal human dermal fibroblast monolayers, Vfe significantly stimulated the expression of collagen I and IV (**Table 4** and **Figure 7**).

## Conclusion

Vfe is an original extract containing nutritional essential elements for the skin cells and the skin microbiome. It acts preferentially by binding to TLR2, which is present at the surface of all skin cells, including keratinocytes, melanocytes, sensitive neurons, Langerhans cells, and DCs. Due to its composition rich in essential elements important for the support of the skin and the microbiome, as well as the stimulation it exerts on the cells *via* the TLRs, Vfe has an optimal bio-affinity with the skin and mimics action shared with beneficial skin microorganisms.

Indeed, Vfe has been shown *in vitro* to have specific efficacy in modulating immunity, inducing natural defence mechanisms, and increasing skin resistance. Furthermore, topical applications of Vfe *in vivo* were shown to have a direct effect on the skin to improve symptoms of seborrheic dermatitis and atopic eczema by soothing the skin, restoring the skin barrier, stimulating AMP, and thus rebalancing skin microbiota.

As the local mode of action of Vfe on the skin is similar to that of probiotics in the gut, especially the ability to modulate immunity and inflammation, stimulate skin defences, and improve the skin barrier/structural dermis, Vfe may thus be considered as non-replicative probiotic fractions.

## Author Contributions

AG designed and implemented the research, analyse the results, discussed the results, and wrote the manuscript. ML discussed the results and contributed to the final manuscript. SC discussed the results and contributed to the final manuscript. FJ and DF designed some studies, performed some experiments, and contributed to the final manuscript. LB designed and implemented the research, discussed the results, and contributed to the final manuscript. All authors contributed to the article and approved the submitted version.

## Funding

All studies were funded by L’Oréal Research & Innovation.

## Conflict of Interest

All authors are employees of L’Oréal Research & Innovation, France.

## Publisher’s Note

All claims expressed in this article are solely those of the authors and do not necessarily represent those of their affiliated organizations, or those of the publisher, the editors and the reviewers. Any product that may be evaluated in this article, or claim that may be made by its manufacturer, is not guaranteed or endorsed by the publisher.

## References

[B1] Amati L. Pepe M. Passeri M. E. Mastronardi M. L. Jirillo E. Covelli V. (2006). Toll-Like Receptor Signaling Mechanisms Involved in Dendritic Cell Activation: Potential Therapeutic Control of T Cell Polarization. Curr. Pharm. Design 12, 4247–4254. doi: 10.2174/138161206778743583 17100626

[B2] Ameen A. Abdulridha M. Najeeb A. (2019). Comparative Effectiveness of Probiotics Timing Regimen in Helicobacter Pylori-Induced Peptic Ulcer Disease Patients. J. Pharm. Sci. & Res 11, 75–83.

[B3] Benno Y. He F. Hosoda M. Hashimoto H. Kojima T. Yamazaki K.. (1996). Effects of Lactobacillus GG Yogurt on Human Intestinal Microecology in Japanese Subjects. Nutr. Today 31, 12S. doi: 10.1097/00017285-199611001-00004

[B4] Benyacoub J. Bosco N. Blanchard C. Demont A. Philippe D. Castiel-Higounenc I.. (2014). Immune Modulation Property of Lactobacillus Paracasei NCC2461 (ST11) Strain and Impact on Skin Defences. Beneficial Microbes 5, 129–136. doi: 10.3920/bm2013.0014 24322880

[B5] Bergey D. H. Holt J. G. (1994). 9th eds. Bergey's Manual of Determinative Bacteriology (Baltimore: The Williams and Wilkins Co), p. 837–53.

[B6] Bergonzelli G. E. Blum S. Brussow H. Corthésy-Theulaz I. (2005). Probiotics as a Treatment Strategy for Gastrointestinal Diseases? Digestion 72, 57–68. doi: 10.1159/000087638 16113543

[B7] Bernet-Camard M. F. Liévin V. Brassart D. Neeser J. R. Servin A. L. Hudault S. (1997). The Human Lactobacillus Acidophilus Strain LA1 Secretes a Nonbacteriocin Antibacterial Substance(s) Active *In Vitro* and *In Vivo* . Appl. Environ. Microbiol. 63, 2747–2753. doi: 10.1128/aem.63.7.2747-2753.1997 9212421 PMC168570

[B8] Boisnic S. Branchet-Gumila M. C. Coutanceau C. (2003). Inhibitory Effect of Oatmeal Extract Oligomer on Vasoactive Intestinal Peptide-Induced Inflammation in Surviving Human Skin. Int. J. Tissue Reactions 25, 41–46. 14518591

[B9] Borruel N. Casellas F. Antolín M. Llopis M. Carol M. Espíin E.. (2003). Effects of Nonpathogenic Bacteria on Cytokine Secretion by Human Intestinal Mucosa. Am. J. Gastroenterol. 98, 865–870. doi: 10.1111/j.1572-0241.2003.07384.x 12738469

[B10] Braff M. H. Di Nardo A. Gallo R. L. (2005). Keratinocytes Store the Antimicrobial Peptide Cathelicidin in Lamellar Bodies. J. Invest. Dermatol. 124, 394–400. doi: 10.1111/j.0022-202X.2004.23443.x 15675959

[B11] Brandi J. Cheri S. Manfredi M. Di Carlo C. Vita Vanella V. Federici F.. (2020). Exploring the Wound Healing, Anti-Inflammatory, Anti-Pathogenic and Proteomic Effects of Lactic Acid Bacteria on Keratinocytes. Sci. Rep. 10 (1), 11572. doi: 10.1038/s41598-020-68483-4 32665600 PMC7360600

[B12] Cebra J. J. (1999). Influences of Microbiota on Intestinal Immune System Development. Am. J. Clin. Nutr. 69, 1046s–1051s. doi: 10.1093/ajcn/69.5.1046s 10232647

[B13] Chen Y. E. Fischbach M. A. Belkaid Y. (2018). Skin Microbiota–Host Interactions. Nature 553, 427–436. doi: 10.1038/nature25177 29364286 PMC6075667

[B14] Christensen H. R. Frøkiaer H. Pestka J. J. (2002). Lactobacilli Differentially Modulate Expression of Cytokines and Maturation Surface Markers in Murine Dendritic Cells. J. Immunol. (Baltimore Md.: 1950) 168, 171–178. doi: 10.4049/jimmunol.168.1.171 11751960

[B15] Coconnier M. H. Bernet M. F. Kernéis S. Chauvière G. Fourniat J. Servin A. L. (1993). Inhibition of Adhesion of Enteroinvasive Pathogens to Human Intestinal Caco-2 Cells by Lactobacillus Acidophilus Strain LB Decreases Bacterial Invasion. FEMS Microbiol. Lett. 110, 299–305. doi: 10.1111/j.1574-6968.1993.tb06339.x 8354463

[B16] Coconnier M. H. Lievin V. Hemery E. Servin A. L. (1998). Antagonistic Activity Against Helicobacter Infection *In Vitro* and *In Vivo* by the Human Lactobacillus Acidophilus Strain LB. Appl. Environ. Microbiol. 64, 4573–4580. doi: 10.1128/aem.64.11.4573-4580.1998 9797324 PMC106686

[B17] Contreras S. Sagory-Zalkind P. Blanquart H. Iltis A. Morand S. (2017). Complete Genome Sequence of Vitreoscilla Filiformis (ATCC 15551), Used as a Cosmetic Ingredient. Genome Announc. 5, e00913–17. doi: 10.1128/genomeA.00913-17 PMC5571427 28839041

[B18] Daliri E. B. Ofosu F. K. Xiuqin C. Chelliah R. Oh D. (2021). Probiotic Effector Compounds: Current Knowledge and Future Perspectives. Front. Microbiol. 12, 655705. doi: 10.3389/fmicb.2021.655705 33746935 PMC7965967

[B19] Deshayes N. Bloas F. Boissout F. Lecardonnel J. Paris M. (2018). 3d *In Vitro* Model of the Re-Epithelialization Phase in the Wound-Healing Process. Exp. Dermatol. 27, 460–462. doi: 10.1111/exd.13390 28603853

[B20] Dhillon P. Singh K. (2020). Therapeutic Applications of Probiotics in Ulcerative Colitis: An Updated Review. PharmaNutrition 13, 100194. doi: 10.1016/j.phanu.2020.100194

[B21] Drago L. (2019). Probiotics and Colon Cancer. Microorganisms 7, 1–11. doi: 10.3390/microorganisms7030066 PMC6463067 30823471

[B22] Du X. Wang L. Wu S. Yuan L. Tang S. Xiang Y.. (2019). Efficacy of Probiotic Supplementary Therapy for Asthma, Allergic Rhinitis, and Wheeze: A Meta-Analysis of Randomized Controlled Trials. Allergy Asthma Proc. 40, 250–260. doi: 10.2500/aap.2019.40.4227 31262380

[B23] Fang Y. Brent Polk D. (2020). Probiotics and Probiotic-Derived Functional Factors—Mechanistic Insights Into Applications for Intestinal Homeostasis. Front. Immunol. 11, 1428. doi: 10.3389/fimmu.2020.01428 32719681 PMC7348054

[B24] FAO/WHO Food and Agriculture Organization of the United Nations/World Health Organization. (2001). Joint FAO/WHO Expert Consultation on Evaluation of Health and Nutritional Properties of Probiotics in Food Including Powder Milk With Live Lactic Acid Bacteria. Available at: http://www.fao.org/publications/card/en/c/7c102d95-2fd5-5b22-8faf-f0b2e68dfbb6/.

[B25] Franca K. (2021). Topical Probiotics in Dermatological Therapy and Skincare: A Concise Review. Dermatol. Ther. (Heidelb) 11, 71–77. doi: 10.1007/s13555-020-00476-7 33340341 PMC7859136

[B26] Fu C. Chen J. Lu J. Yi L. Tong X. Kang L.. (2020). Roles of Inflammation Factors in Melanogenesis. Mol. Med. Rep. 21, 1421–1430. doi: 10.3892/mmr.2020.10950 32016458 PMC7002987

[B27] Fuller R. (1989). Probiotics in Man and Animals. J. Appl. Bacteriol. 66, 365–378. doi: 10.1111/j.1365-2672.1989.tb05105.x 2666378

[B28] Gordon S. (2008). Elie Metchnikoff: Father of Natural Immunity. Eur. J. Immunol. 38, 3257–3264. doi: 10.1002/eji.200838855 19039772

[B29] Grice E. A. Kong H. H. Conlan S. Deming C. B. Davis J. Young A. C.. (2009). Topographical and Temporal Diversity of the Human Skin Microbiome. Science 324, 1190–1192. doi: 10.1126/science.1171700 19478181 PMC2805064

[B30] Grice E. A. Segre J. A. (2011). The Skin Microbiome. Nat. Rev. Microbiol. 9, 244–253. doi: 10.1038/nrmicro2537 21407241 PMC3535073

[B31] Gueniche A. Bastien P. Ovigne J. M. Kermici M. Courchay G. Chevalier V.. (2010). Bifidobacterium Longum Lysate, a New Ingredient for Reactive Skin. Exp. Dermatol. 19, e1–e8. doi: 10.1111/j.16000625.2009.00932.x 19624730

[B32] Gueniche A. Cathelineau A. C. Bastien P. Esdaile J. Martin R. Queille-Roussel C.. (2008a). Vitreoscilla Filiformis Biomass Improves Seborrheic Dermatitis. J. Eur. Acad. Dermatol. Venereol. JEADV 22, 1014–1015. doi: 10.1111/j.1468-3083.2007.02508.x 18194241

[B33] Gueniche A. Dahel K. Bastien P. Martin R. Nicolas J. F. Breton L. (2008b). Vitreoscilla Filiformis Bacterial Extract to Improve the Efficacy of Emollient Used in Atopic Dermatitis Symptoms. J. Eur. Acad. Dermatol. Venereol. JEADV 22, 746–747. doi: 10.1111/j.1468-3083.2007.02428.x 18482031

[B34] Gueniche A. Hennino A. Goujon C. Dahel K. Bastien P. Martin R.. (2006). Improvement of Atopic Dermatitis Skin Symptoms by Vitreoscilla Filiformis Bacterial Extract. Eur. J. Dermatol. 16, 380–384. 16935794

[B35] Gueniche A. Knaudt B. Schuck E. Volz T. Bastien P. Martin R.. (2008c). Effects of Nonpathogenic Gram-Negative Bacterium Vitreoscilla Filiformis Lysate on Atopic Dermatitis: A Prospective, Randomized, Double-Blind, Placebo-Controlled Clinical Study. Br. J. Dermatol. 159, 1357–1363. doi: 10.1111/j.1365-2133.2008.08836.x 18795916

[B36] Gueniche A. Philippe D. Bastien P. Reuteler G. Blum S. Castiel-Higounenc I.. (2014). Randomised Double-Blind Placebo-Controlled Study of the Effect of Lactobacillus Paracasei NCC 2461 on Skin Reactivity. Beneficial Microbes 5, 137–145. doi: 10.3920/bm2013.0001 24322879

[B37] Guslandi M. Mezzi G. Sorghi M. Testoni P. A. (2000). Saccharomyces Boulardii in Maintenance Treatment of Crohn's Disease. Digest. Dis. Sci. 45, 1462–1464. doi: 10.1023/a:1005588911207 10961730

[B38] Han N. Jia L. Guo L. Su Y. Luo Z. Du J.. (2020). Balanced Oral Pathogenic Bacteria and Probiotics Promoted Wound Healing *via* Maintaining Mesenchymal Stem Cell Homeostasis. Stem Cell Res. Ther. 11, 61. doi: 10.1186/s13287-020-1569-2 32059742 PMC7023757

[B39] Harder J. Meyer-Hoffert U. Wehkamp K. Schwichtenberg L. Schröder J. M. (2004). Differential Gene Induction of Human Beta-Defensins (hBD-1, -2, -3, and -4) in Keratinocytes Is Inhibited by Retinoic Acid. J. Invest. Dermatol. 123, 522–529. doi: 10.1111/j.0022-202X.2004.23234.x 15304092

[B40] Hill C. Guarner F. Reid G. Gibson G. R. Merenstein D. J. Pot B.. (2014). Expert Consensus Document. The International Scientific Association for Probiotics and Prebiotics Consensus Statement on the Scope and Appropriate Use of the Term Probiotic. Nat. Rev. Gastroenterol. Hepatol. 11, 506–514. doi: 10.1038/nrgastro.2014.66 24912386

[B41] Isolauri E. (2001). Probiotics in the Prevention and Treatment of Allergic Disease. Pediatr. Allergy Immunol. 12 Suppl 14, 56–59. doi: 10.1034/j.1399-3038.2001.121413.x 11380901

[B42] Isolauri E. Sütas Y. Kankaanpää P. Arvilommi H. Salminen S. (2001). Probiotics: Effects on Immunity. Am. J. Clin. Nutr. 73, 444s–450s. doi: 10.1093/ajcn/73.2.444s 11157355

[B43] Jung Y. O. Jeong H. Cho Y. Lee E. O. Jang H. W. Kim J.. (2019). Lysates of a Probiotic, *Lactobacillus Rhamnosus*, Can Improve Skin Barrier Function in a Reconstructed Human Epidermis Model. Int. J. Mol. Sci. 20 (17), 1–12. doi: 10.3390/ijms20174289 PMC6747158 31480681

[B44] Kalliomäki M. Salminen S. Arvilommi H. Kero P. Koskinen P. Isolauri E. (2001). Probiotics in Primary Prevention of Atopic Disease: A Randomised Placebo-Controlled Trial. Lancet 357, 1076–1079. doi: 10.1016/s0140-6736(00)04259-8 11297958

[B45] Kawai K. Shimura H. Minagawa M. Ito A. Tomiyama K. Ito M. (2002). Expression of Functional Toll-Like Receptor 2 on Human Epidermal Keratinocytes. J. Dermatol. Sci. 30, 185–194. doi: 10.1016/s0923-1811(02)00105-6 12443841

[B46] Kempkes C. Guéniche A. Cevikbas F. Steinhoff M. (2012). Vitreoscilla Filiformis Extract Decrease Pruritic Mediators Involved in Skin Disease, European Society for Dermatological Research, Venice, Italy. J. Invest. Dermatol. 32 (2), 375–384. doi: 10.1038/jid.2011.314

[B47] Khare A. Thorat G. Bhimte A. Yadav V. (2018). Mechanism of Action of Prebiotic and Probiotic. J. Entomol Zool Stud. 6, 51–53.

[B48] Kistowska M. Fenini G. Jankovic D. Feldmeyer L. Kerl K. Bosshard P.. (2014). Malassezia Yeasts Activate the NLRP3 Inflammasome in Antigen-Presenting Cells *via* Syk-Kinase Signalling. Exp. Dermatol. 23 (12), 884–889. doi: 10.1111/exd.12552 25267545

[B49] Langhendries J. P. Detry J. Van Hees J. Lamboray J. M. Darimont J. Mozin M. J.. (1995). Effect of a Fermented Infant Formula Containing Viable Bifidobacteria on the Fecal Flora Composition and pH of Healthy Full-Term Infants. J. Pediatr. Gastroenterol. Nutr. 21, 177–181. doi: 10.1097/00005176-199508000-00009 7472904

[B50] Li L. Han Z. Niu X. Zhang G. Jia Y. Zhang S.. (2019). Probiotic Supplementation for Prevention of Atopic Dermatitis in Infants and Children: A Systematic Review and Meta-Analysis. Am. J. Clin. Dermatol. 20, 367–377. doi: 10.1007/s40257-018-0404-3 30465329

[B51] Li L. Jun H. Dawei Z. Jammayrac O. Bastien P. Carpentier M.. (2006). Evaluation of the Efficacy and Skin Tolerance of a Cream Containing 1% Vitreoscilla Filiformis Extract Applied on Chinese Women With Sensitive Skin. Chin. J. Med. Aesthetics Cosmetol. 12, 195–197.

[B52] Lim H. Y. Jeong D. Park S. H. Shin K. K. Hong Y. H. Kim E.. (2020). Antiwrinkle and Antimelanogenesis Effects of Tyndallized Lactobacillus Acidophilus. Int. J. Mol. Sci. 21 (5), 1–16. doi: 10.3390/ijms21051620 PMC7084287 32120828

[B53] Liu D.-M. Guo J. Zeng X.-A. Sun D.-W. Brennan C. S. Zhou Q.-X.. (2017). The Probiotic Role of Lactobacillus Plantarum in Reducing Risks Associated With Cardiovascular Disease. Int. J. Food Sci. Technol. 52, 127–136. doi: 10.1111/ijfs.13234

[B54] Lopes E. G. Moreira D. A. Gullon P. Gullon B. Cardelle-Cobas A. Tavaria F. K. (2017). Topical Application of Probiotics in Skin: Adhesion, Antimicrobial and Antibiofilm *In Vitro* Assays. J. Appl. Microbiol. 122, 450–461. doi: 10.1111/jam.13349 27862685

[B55] MacFarland C. (1999). “The Human Colonic Microbiota,” in Nutrition and Health. Eds. Gibson C. M. R G.. (New York City, USA: Kluwer Academic Publisher), 1–25.

[B56] Magerl M. Lammel V. Siebenhaar F. Zuberbier T. Metz M. Maurer M. (2008). Non-Pathogenic Commensal Escherichia Coli Bacteria can Inhibit Degranulation of Mast Cells. Exp. Dermatol. 17, 427–435. doi: 10.1111/j.1600-0625.2008.00704.x 18331331

[B57] Mahé Y. F. Martin R. Aubert L. Billoni N. Collin C. Pruche F.. (2006). Induction of the Skin Endogenous Protective Mitochondrial MnSOD by Vitreoscilla Filiformis Extract. Int. J. Cosmetic Sci. 28, 277–287. doi: 10.1111/j.1467-2494.2006.00333.x 18489268

[B58] Mahe Y. F. Perez M. J. Tacheau C. Fanchon C. Martin R. Rousset F.. (2013). A New Vitreoscilla Filiformis Extract Grown on Spa Water-Enriched Medium Activates Endogenous Cutaneous Antioxidant and Antimicrobial Defenses Through a Potential Toll-Like Receptor 2/Protein Kinase C, Zeta Transduction Pathway. Clin. Cosmetic Invest. Dermatol. 6, 191–196. doi: 10.2147/ccid.S47324 PMC3770492 24039440

[B59] Mathur H. Beresford T. P. Cotter P. D. (2020). Health Benefits of Lactic Acid Bacteria (LAB) Fermentates. Nutrients 12 (6), 1–12. doi: 10.3390/nu12061679 PMC7352953 32512787

[B60] McFarland C. Evans C. T. Goldstein E. J. C. (2018). Strain-Specificity and Disease-Specificity of Probiotic Efficacy: A Systematic Review and Meta-Analysis. Front. Med. 5, 124. doi: 10.3389/fmed.2018.00124 PMC5949321 29868585

[B61] Medzhitov R. Janeway C. Jr. (2000). The Toll Receptor Family and Microbial Recognition. Trends Microbiol. 8, 452–456. doi: 10.1016/s0966-842x(00)01845-x 11044679

[B62] Midolo P. D. Lambert J. R. Hull R. Luo F. Grayson M. L. (1995). *In Vitro* Inhibition of Helicobacter Pylori NCTC 11637 by Organic Acids and Lactic Acid Bacteria. J. Appl. Bacteriol. 79, 475–479. doi: 10.1111/j.1365-2672.1995.tb03164.x 7592140

[B63] Moayyedi P. Ford A. C. Talley N. J. Cremonini F. Foxx-Orenstein A. E. Brandt L. J.. (2010). The Efficacy of Probiotics in the Treatment of Irritable Bowel Syndrome: A Systematic Review. Gut 59, 325–332. doi: 10.1136/gut.2008.167270 19091823

[B64] Mohan R. Koebnick C. Schildt J. Schmidt S. Mueller M. Possner M.. (2006). Effects of Bifidobacterium Lactis Bb12 Supplementation on Intestinal Microbiota of Preterm Infants: A Double-Blind, Placebo-Controlled, Randomized Study. J. Clin. Microbiol. 44, 4025–4031. doi: 10.1128/jcm.00767-06 16971641 PMC1698302

[B65] Mukhopadhyay S. Herre J. Brown G. D. Gordon S. (2004). The Potential for Toll-Like Receptors to Collaborate With Other Innate Immune Receptors. Immunology 112, 521–530. doi: 10.1111/j.1365-2567.2004.01941.x 15270722 PMC1782521

[B66] Oliveira-Nascimento L. Massari P. Wetzler L. M. (2012). The Role of TLR2 in Infection and Immunity. Front. Immunol. 3, 79. doi: 10.3389/fimmu.2012.00079 22566960 PMC3342043

[B67] Ouwehand A. C. Båtsman A. Salminen S. (2003). Probiotics for the Skin: A New Area of Potential Application? Lett. Appl. Microbiol. 36, 327–331. doi: 10.1046/j.1472-765x.2003.01319.x 12680947

[B68] Peguet-Navarro J. Dezutter-Dambuyant C. Buetler T. Leclaire J. Smola H. Blum S.. (2008). Supplementation With Oral Probiotic Bacteria Protects Human Cutaneous Immune Homeostasis After UV Exposure-Double Blind, Randomized, Placebo Controlled Clinical Trial. Eur. J. Dermatol. 18, 504–511. doi: 10.1684/ejd.2008.0496 18693151

[B69] Pivarcsi A. Bodai L. Réthi B. Kenderessy-Szabó A. Koreck A. Széll M.. (2003). Expression and Function of Toll-Like Receptors 2 and 4 in Human Keratinocytes. Int. Immunol. 15, 721–730. doi: 10.1093/intimm/dxg068 12750356

[B70] Pochapin M. (2000). The Effect of Probiotics on Clostridium Difficile Diarrhea. Am. J. Gastroenterol. 95, S11–S13. doi: 10.1016/s0002-9270(99)00809-6 10634221

[B71] Rao R. K. Samak G. (2013). Protection and Restitution of Gut Barrier by Probiotics: Nutritional and Clinical Implications. Curr. Nutr. Food Sci. 9, 99–107. doi: 10.2174/1573401311309020004 24353483 PMC3864899

[B72] Rautava S. Isolauri E. (2002). The Development of Gut Immune Responses and Gut Microbiota: Effects of Probiotics in Prevention and Treatment of Allergic Disease. Curr. Issues Intestinal Microbiol. 3, 15–22. 12022809

[B73] Reygagne P. Bastien P. Couavoux M. P. Philippe D. Renouf M. Castiel-Higounenc I.. (2017). The Positive Benefit of Lactobacillus Paracasei NCC2461 ST11 in Healthy Volunteers With Moderate to Severe Dandruff. Beneficial Microbes 8, 671–680. doi: 10.3920/bm2016.0144 28789559

[B74] Ringel-Kulka T. Goldsmith J. R. Carroll I. M. Barros S. P. Palsson O. Jobin C.. (2014). Lactobacillus Acidophilus NCFM Affects Colonic Mucosal Opioid Receptor Expression in Patients With Functional Abdominal Pain - a Randomised Clinical Study. Aliment. Pharmacol. Ther. 40, 200–207. doi: 10.1111/apt.12800 24853043 PMC4613798

[B75] Roberfroid M. B. Bornet F. Bouley C. Cummings J. H. (1995). Colonic Microflora: Nutrition and Health. Summary and Conclusions of an International Life Sciences Institute (ILSI) [Europe] Workshop Held in Barcelona, Spain. Nutr. Rev. 53, 127–130. doi: 10.1111/j.1753-4887.1995.tb01535.x 7666984

[B76] Rodrigues K. L. Caputo L. R. Carvalho J. C. Evangelista J. Schneedorf J. M. (2005). Antimicrobial and Healing Activity of Kefir and Kefiran Extract. Int. J. Antimicrob. Agents 25, 404–408. doi: 10.1016/j.ijantimicag.2004.09.020 15848295

[B77] Rolfe R. D. (2000). The Role of Probiotic Cultures in the Control of Gastrointestinal Health. J. Nutr. 130, 396s–402s. doi: 10.1093/jn/130.2.396S 10721914

[B78] Sanders M. E. Benson A. Lebeer S. Merenstein D. J. Klaenhammer T. R. (2018). Shared Mechanisms Among Probiotic Taxa: Implications for General Probiotic Claims. Curr. Opin. Biotechnol. 49, 207–216. doi: 10.1016/j.copbio.2017.09.007 29128720

[B79] Sarker S. A. Sultana S. Fuchs G. J. Alam N. H. Azim T. Brüssow H.. (2005). Lactobacillus Paracasei Strain ST11 Has No Effect on Rotavirus But Ameliorates the Outcome of Nonrotavirus Diarrhea in Children From Bangladesh. Pediatrics 116, e221–e228. doi: 10.1542/peds.2004-2334 15995003

[B80] Sawada J. Morita H. Tanaka A. Salminen S. He F. Matsuda H. (2007). Ingestion of Heat-Treated Lactobacillus Rhamnosus GG Prevents Development of Atopic Dermatitis in NC/Nga Mice. Clin. Exp. Allergy J. Br. Soc. Allergy Clin. Immunol. 37, 296–303. doi: 10.1111/j.1365-2222.2006.02645.x 17250703

[B81] Schepper J. D. Collins F. L. Rios-Arce N. D. Raehtz S. Schaefer L. Gardinier J. D.. (2019). Probiotic Lactobacillus Reuteri Prevents Postantibiotic Bone Loss by Reducing Intestinal Dysbiosis and Preventing Barrier Disruption. J. Bone Mineral Res. Off. J. Am. Soc. Bone Mineral Res. 34, 681–698. doi: 10.1002/jbmr.3635 PMC6557403 30690795

[B82] Schmidt T. M. Arieli B. Cohen Y. Padan E. Strohl W. R. (1987). Sulfur Metabolism in Beggiatoa Alba. J. Bacteriol. 169, 5466–5472. doi: 10.1128/jb.169.12.5466-5472.1987 3316186 PMC213973

[B83] Serhan C. N. Levy B. D. (2018). Resolvins in Inflammation: Emergence of the Pro-Resolving Superfamily of Mediators. J. Clin. Invest. 128, 2657–2669. doi: 10.1172/JCI97943 29757195 PMC6025982

[B84] Stahl D. A. Lane D. J. Olsen G. J. Heller D. J. Schmidt T. M. Pace N. R. (1987). Phylogenetic Analysis of Certain Sulfide-Oxidizing and Related Morphologically Conspicuous Bacteria by 5S Ribosomal Ribonucleic Acid Sequences. Int. J. System. Evol. Microbiol. 37, 116–122. doi: 10.1099/00207713-37-2-116

[B85] Strohl W. R. (2005). “Genus XII. Vitreoscilla,” in Bergey’s Manual of Systematic Bacteriology, vol. 2 . Eds. Staley J. T. Boone D. R. Brenner D. J. (New York: Springer), 851–858. The Proteobacteria.

[B86] Strohl W. Schmidt T. Lawry N. Mezzino M. Larkin J. (1986). Characterization of Vitreoscilla Beggiatoides and Vitreoscilla Filiformis Sp. Nov., Nom. Rev., and Comparison With Vitreoscilla Stercoraria and Beggiatoa Alba. Int. J. System. Bacteriol. 36, 302–313. doi: 10.1099/00207713-36-2-302

[B87] Sugimoto M. A. Vago J. P. Perretti M. Teixeira M. M. (2019). Mediators of the Resolution of the Inflammatory Response. Trends Immunol. 40, 212–227. doi: 10.1016/j.it.2019.01.007 30772190

[B88] Sumikawa Y. Asada H. Hoshino K. Azukizawa H. Katayama I. Akira S.. (2006). Induction of Beta-Defensin 3 in Keratinocytes Stimulated by Bacterial Lipopeptides Through Toll-Like Receptor 2. Microbes Infect. 8, 1513–1521. doi: 10.1016/j.micinf.2006.01.008 16697678

[B89] Suter M. M. Schulze K. Bergman W. Welle M. Roosje P. Muller E. J. (2009). The Keratinocyte in Epidermal Renewal and Defence. Veter. Dermatol. 20, 515–532. doi: 10.1111/j.1365-3164.2009.00819.x 20178490

[B90] Szajewska H. Guarino A. Hojsak I. Indrio F. Kolacek S. Shamir R.. (2014). Use of Probiotics for Management of Acute Gastroenteritis: A Position Paper by the ESPGHAN Working Group for Probiotics and Prebiotics. J. Pediatr. Gastroenterol. Nutr. 58, 531–539. doi: 10.1097/mpg.0000000000000320 24614141

[B91] Urbano P. Aguirre-Gamboa R. Ashikov A. Bennievan Heeswijk B. Krippner-Heidenreich A. Tijssen H.. (2018). TNF-α–Induced Protein 3 (TNFAIP3)/A20 Acts As a Master Switch in TNF-α Blockade–Driven IL-17A Expression. J. All. Clin. Immunol 142, 517–529. doi: 10.1016/j.jaci.2017.11.024 29248493

[B92] Volz T. Skabytska Y. Guenova E. Chen K. M. Frick J. S. Kirschning C. J.. (2014). Nonpathogenic Bacteria Alleviating Atopic Dermatitis Inflammation Induce IL-10-Producing Dendritic Cells and Regulatory Tr1 Cells. J. Invest. Dermatol. 134, 96–104. doi: 10.1038/jid.2013.291 23812300

[B93] von der Weid T. Bulliard C. Schiffrin E. J. (2001). Induction by a Lactic Acid Bacterium of a Population of CD4(+) T Cells With Low Proliferative Capacity That Produce Transforming Growth Factor Beta and Interleukin-10. Clin. Diagn. Lab. Immunol. 8, 695–701. doi: 10.1128/cdli.8.4.695-701.2001 11427413 PMC96129

[B94] White J. S. Hoper M. Parks R. W. Clements W. D. Diamond T. Bengmark S. (2006). The Probiotic Bacterium Lactobacillus Plantarum Species 299 Reduces Intestinal Permeability in Experimental Biliary Obstruction. Lett. Appl. Microbiol. 42, 19–23. doi: 10.1111/j.1472-765X.2005.01800.x 16411914

